# Virtual reality-induced motor function of the upper extremity and brain activation in stroke: study protocol for a randomized controlled trial

**DOI:** 10.3389/fneur.2023.1094617

**Published:** 2023-04-17

**Authors:** Jie Shen, Xudong Gu, Jianming Fu, Yunhai Yao, Yan Li, Ming Zeng, Zhixiang Liu, Cao Lu

**Affiliations:** Center of Rehabilitation Medicine, The Second Hospital of Jiaxing, The Second Affiliated Hospital of Jiaxing University, Jiaxing, Zhejiang, China

**Keywords:** stroke, virtual reality, upper extremity, rehabilitation, fMRI, EEG

## Abstract

**Background:**

The benefits of virtual reality (VR)-based rehabilitation were reported in patients after stroke, but there is insufficient evidence about how VR promotes brain activation in the central nervous system. Hence, we designed this study to explore the effects of VR-based intervention on upper extremity motor function and associated brain activation in stroke patients.

**Methods/design:**

In this single-center, randomized, parallel-group clinical trial with a blinded assessment of outcomes, a total of 78 stroke patients will be assigned randomly to either the VR group or the control group. All stroke patients who have upper extremity motor deficits will be tested with functional magnetic resonance imaging (fMRI), electroencephalography (EEG), and clinical evaluation. Clinical assessment and fMRI will be performed three times on each subject. The primary outcome is the change in performance on the Fugl-Meyer Assessment Upper Extremity Scale (FMA-UE). Secondary outcomes are functional independence measure (FIM), Barthel Index (BI), grip strength, and changes in the blood oxygenation level-dependent (BOLD) effect in the ipsilesional and contralesional primary motor cortex (M1) on the left and right hemispheres assessed with resting-state fMRI (rs-fMRI), task-state fMRI (ts-fMRI), and changes in EEG at the baseline and weeks 4 and 8.

**Discussion:**

This study aims to provide high-quality evidence for the relationship between upper extremity motor function and brain activation in stroke. In addition, this is the first multimodal neuroimaging study that explores the evidence for neuroplasticity and associated upper motor function recovery after VR in stroke patients.

**Clinical trial registration:**

Chinese Clinical Trial Registry, identifier: ChiCTR2200063425.

## Introduction

A stroke, also known as a transient ischemic attack or cerebrovascular accident, occurs when a blood vessel suddenly ruptures or a blood clot becomes blocked, interrupting the supply of oxygen and nutrients to the brain tissue and causing brain tissue damage (1). The annual stroke mortality rate is ~1.57 million in China with a trend toward increasing stroke recurrence and mortality (2). The long-lasting residual impairments and upper extremity dysfunctions typically influence the daily activity and quality of life of a substantial number of stroke survivors. The prevalence of upper extremity impairment is present at ~50–80% in the acute phase (3) and 40–50% in the chronic stroke phase (4). The recovery of motor function in the upper extremities has a significant impact on the ability of stroke patients to live independently.

The field of neurological rehabilitation has begun to apply VR to improve the joint mobility, speed, and precision of extremities in post-stroke hemiplegia patients (5). Compared with conventional rehabilitation, the value of VR in the rehabilitation of motor dysfunction is creating an enhanced environment, facilitating task-specific training, and providing multimodal feedback to augment functional recovery (6). A meta-analysis of 2,470 stroke patients found that VR as an aid to upper extremity motor function helped improve upper extremity motor function and daily living ability (7). On this basis, by incorporating gamification features into rehabilitation, VR also helps to achieve an increased training dose, increased intensity, and increased patient compliance (8, 9). In addition, small sample studies have confirmed the effectiveness of VR as an adjunct to improving upper extremity motor function in stroke patients (9). At present, increasing research has paid attention to VR in the rehabilitation of upper extremity motor dysfunction after stroke and gradually applied brain neuroimaging technology to explore the mechanism of action, which is expected to make effective progress in the future (10).

Functional recovery after brain injury is largely dominated by neuroplasticity, which is a dynamic adaptation of the structure and function of the central nervous system throughout its life cycle (11). The connotation of neuroplasticity involves the axonal remodeling of cortical circuits and the rearrangement of cortical mapping that occurs with disorder recovery (12, 13). The current understanding based on neuroplasticity has important clinical significance for stroke rehabilitation research. The use of intensive, frequent, and obvious task-specific activities should be encouraged in order to support experience-dependent neuroplasticity and functional recovery. VR can enrich the training environment by participating in sensory, cognitive, and perceptual-motor pathways, and it is better able to provide the key components of neuroplasticity described above to support functional recovery outcomes than traditional rehabilitation interventions (14).

Vision is a powerful signal of the sensorimotor center, and visual motor training in virtual reality environments (VE) is based on stimulation of the motor processing system, which activates the lower cortex regions of the brain involved in the performance of the action. There is a rich intrahemispheric cortex–cortex projection relationship between the parietal occipital lobe and the movement-related frontal cortex associated with vision and spatial position (15), which verifies that the visual feedback that controls hand activity could be used to promote the activity of specific brain regions. VR technology is conceivable in terms of immersive and repetitive, intensive therapeutic exercises, and its mechanism of action in post-stroke motor dysfunction may be used to induce changes in neural substrates for restoration and compensation *via* diverse and interesting games (16). In addition, cortical recombination is involved in the recovery of motor function, and interspheric inhibition has been confirmed in the VR study of the upper extremities of stroke (17, 18). VR of the upper extremities can achieve a higher degree of cerebral cortical recombination than conventional rehabilitation training, that is, the activation of the laterality index in the healthy hemisphere decreased significantly after training and the activation of the laterality index in the affected hemisphere increased significantly (17).

Although many reviews of VR interventions have shown positive improvements in stroke rehabilitation, most studies have focused on the impact of VR intervention-based rehabilitation on the treatment of dysfunction, and only a few studies have focused on changes in the central nervous system in stroke patients under VR intervention. With high spatial resolution, fMRI might detect patterns of brain excitation during motor work or rest by using the blood oxygen level-dependent signal as an alternative indicator of neural activity, but the precision of fMRI is restricted. EEG technology detects electrical activity for the purposes of providing enhanced efficiency data and identifying critical neural substrates that underlie certain functional deficits. In the examination of patients with stroke, quantitative EEG measures demonstrated not only clinical value but also multidimensional repeatability and reliability (19). The use of multimodal neuroimaging to obtain more comprehensive, rich, accurate, and reliable brain information provides new monitoring means for the description of neural dynamic processes (20). Neuroimaging techniques could indirectly describe angiogenesis and neuroplasticity and provide quantifiable indicators and responses to therapeutic interventions for stroke (21). Synchronous EEG-fMRI could simultaneously collect electrophysiological and blood oxygen metabolism signals in brain activities, combining the advantages of the two, which not only provides the feasibility of fusion for the study of the two modes (22) but also provides a new means for understanding the description of neural dynamic processes, which plays an irreplaceable role. Therefore, the primary goal of this study is to explore the effects of VR intervention on upper extremity motor function and associated brain activation in stroke patients. Multimodal neuroimaging characteristics that could be used as indicators of a better response to VR were also investigated.

## Methods and analysis

### Design and setting

**Figure 1:**
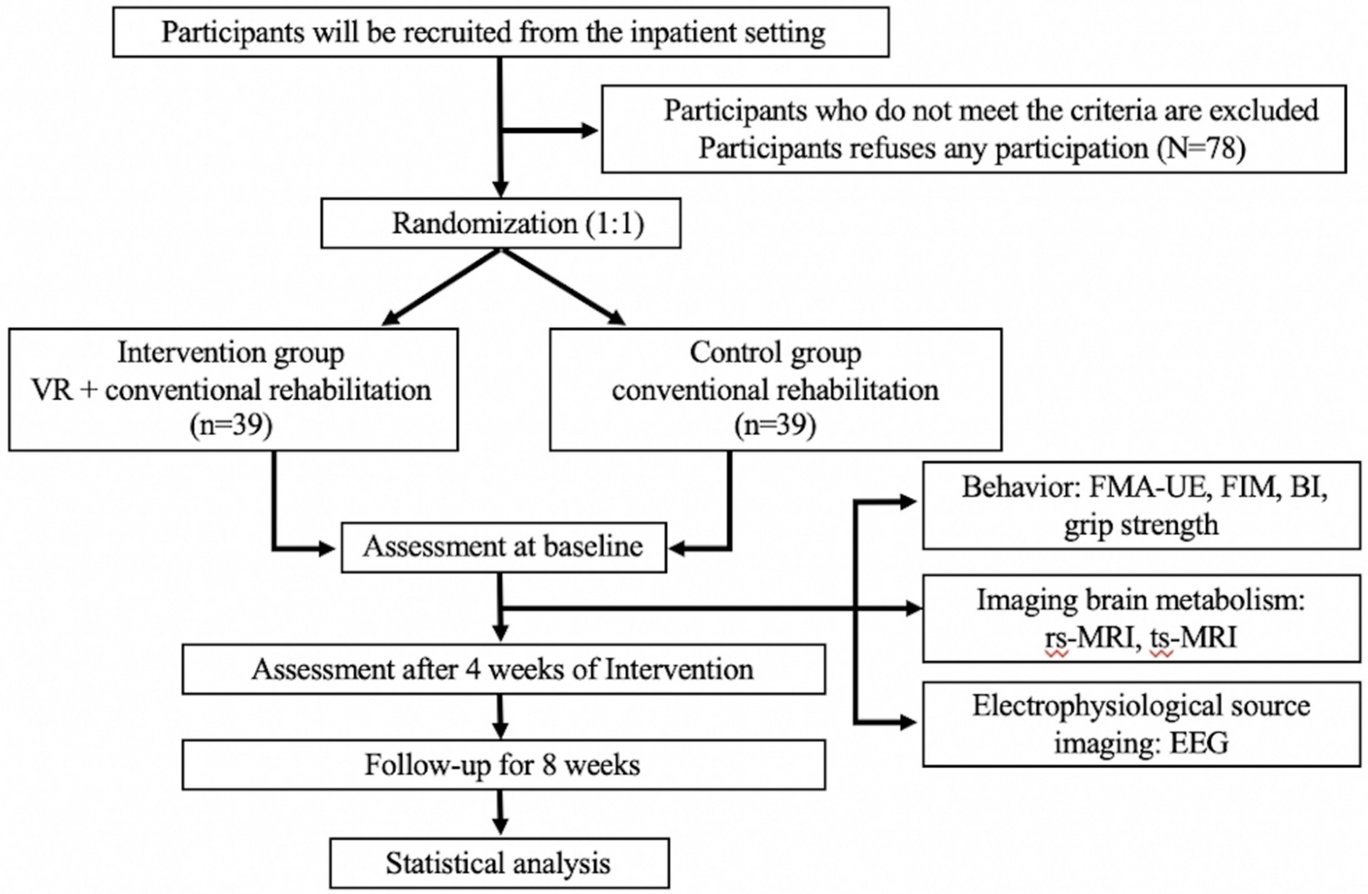
Flow chart of the trial.

This is a prospective, randomized controlled trial conducted by the Second Hospital of Jiaxing. A total of 78 patients who meet the inclusion and exclusion criteria will be randomly divided into two groups. The intervention group will receive VR training and conventional rehabilitation treatment, and the control group will receive conventional rehabilitation treatment only. All treatments will be provided five times per week for 4 weeks. The primary outcome is the degree of restored upper extremity motor function, which is evaluated by the Fugl-Meyer Assessment Upper Extremity Scale (FMA-UE). Secondary outcomes are neuroimaging outcomes, which are measured by resting-state functional magnetic resonance imaging (rs-fMRI), task-state functional magnetic resonance imaging (ts-fMRI), and electroencephalography (EEG). All outcomes will be evaluated at baseline and weeks 4 and 8. The study's flow chart is shown in Figure 1. The Standard Protocol Items: Recommendations for Interventional Trials (SPIRIT) checklist (23) is detailed in an additional document available upon request (Supplementary material), and the schedule of this study is shown in Table 1.

**Table 1:** Process chart of the trial.

**Timepoint**	**Study period**			
	**Enrollment-1 week**	**Baseline 0 week**	**Treatment phase 4 weeks**	**Follow-up phase 8 weeks**
**Enrollment**				
Eligibility screen	✓			
Demographic	✓			
**Characteristics**				
Informed consent	✓			
Medical history	✓			
Merger disease	✓			
Randomization		✓		
**Interventions**				
Intervention group		✓	✓	
Control group		✓	✓	
**Assessments**				
FMA-UE		✓	✓	✓
FIM		✓	✓	✓
BI		✓	✓	✓
Grip strength		✓	✓	✓
rs-fMRI		✓	✓	✓
ts-fMRI		✓	✓	✓
EEG		✓	✓	✓

FMA-UE, Fugl-Meyer assessment for the upper extremity; FIM, functional independence measure; BI, barthel index; rs-fMRI, resting-state fMRI; ts-fMRI, task-state fMRI; EEG, electroencephalography.

### Recruitment of participants

This is the version of the protocol, dated 6 September 2022. Patients who meet the criteria will be recruited through outpatient and inpatient systems and advertisements. The recruitment began on January 2023 and is expected to end in December 2023. The trial registration number of the study is ChiCTR2200063425.

### Inclusion criteria

Participants who meet the following criteria are included:

(1) All meet the diagnostic criteria for stroke formulated by the 4th National Conference on Cerebrovascular Disease in 1995.
(2) The ischemic cortical or subcortical stroke occurs for the first time.
(3) The level of upper extremity motor function can execute at least four of the seven tasks in daily life as part of the Jebsen–Taylor test.
(4) Age ≥ 18 years.
(5) Normal hearing and vision and smooth communication.
(6) No severe cognitive impairment and also mini-mental state examination (MMSE) with a score of 16 or above.

### Exclusion criteria

Participants with the following conditions are excluded:

(1) Cerebral hemorrhage, subarachnoid hemorrhage, venous sinus thrombosis, transient ischemic attack, progressive stroke, or reversible ischemic attack.
(2) Patients with a history of mental disorder or the use of antipsychotic medication or obvious suicidal tendencies.
(3) Women who are pregnant.
(4) Lesions located in both cerebral hemispheres, cerebellum, or brainstem.
(5) Patients who have severe heart, liver, kidney, and other medical diseases or tumors.
(6) Patients who have a history of metal implantation in the past and cannot cooperate with the MRI examination.

### Randomization

The random allocation cards will be generated by an independent researcher using computer-generated random numbers. The qualifying patients who can be enrolled are then given the randomization sequence, which is then placed in secret, opaque envelopes.

### Blinding

Due to the nature of the VR training and the technical constraints, we are unable to undertake a double-blind study design. However, evaluators and statisticians of the outcomes will be unaware of the assignments. Patients will be treated independently to prevent communication, and only certified and experienced therapists will be allowed to conduct the VR training sessions. Therapists supply patients, evaluators, or statisticians with whatever information they may need on the allocation. An independent researcher will carry out analysis tasks during the data management and statistical analysis processes.

### Interventions

All patients will receive conventional rehabilitation, which includes joint loosening training, passive stretching, relaxation training, stepping training, and endurance training, whereas the VR group will receive 30 min of conventional rehabilitation and 30 min of VR training. The control group will receive 30 min of conventional rehabilitation and 30 min of upper extremity function exercise. Rehabilitation will be done five times per week for 4 weeks in separate rooms for a total of 20 sessions in the hospital. All treatments will be performed by licensed therapists with at least 3 years of practice experience. The interventions of the two groups are as follows.

### Interventions group

VR training and conventional rehabilitation will be used in the intervention group for 30 min. The VR system hardware is mainly composed of the following four parts: Wii host, remote controller (RC), complementary metal oxide semiconductor (CMOS) infrared sensor, and liquid crystal display (LCD). The RC uses embedded acceleration sensors to detect the direction, speed, and acceleration of the user's upper extremities and hands in three-dimensional space. CMOS infrared sensors capture and reproduce the patient's motion trajectory on a display by receiving infrared signals from both ends of the receiving optical sensor rod. Training programs include tennis, table tennis, and kitchen cooking. Upper extremity movements include shoulder flexion, stretching (tennis), shoulder internal rotation, external rotation (tennis); elbow flexion, stretching (tennis, table tennis, and kitchen cooking); and radial joints before and after rotation (tennis, table tennis, and kitchen cooking). During Wii game training, the patient will stay seated and use the affected upper extremity for game training, and if the patient is unable to hold the remote controller, an elastic bandage is used to fix the controller on the affected upper hand. The existing evidence on rehabilitation of the upper extremity after stroke proposes that constraining or forced use of 90% of the training time is devoted to using the non-paretic upper extremity with a mitt to encourage the use of the more affected extremity (24). Taking into account the previous level of stroke patients, the degree of the Wii training difficulty will be divided into several degrees in all courses. To prevent weariness during training, the therapist will give the participants a thorough explanation of the Wii game training procedure.

### Control group

Control group participants will receive conventional rehabilitation training and upper extremity function exercises. Upper extremity function exercises include passive and active assistance, active training of the upper extremities on the affected side, training in pushing scrub boards on the upper extremities on the affected side, training in grasping and opening the upper extremities on the affected side, training of a range of motion, muscle strength, sitting balance ability with the help of tools, such as scrub boards, rollers, and sticks, using screws, and other training of hand fine movements, and sitting balance ability using tools such as a chair.

### Sample size

The objective of this study is to explore VR and conventional rehabilitation for upper extremity motor function after a stroke and to calculate the sample size by using G-Power (version 3.1) software. The effect size f is expected to be at least 0.25, and since the power is 0.80, the protocol should include 65 participants. Considering an expected 15% dropout rate, the sample size is increased to 78 participants.

### Outcome measures

Participants will be evaluated at baseline and weeks 4 and 8. All evaluations will be conducted by researchers who will be blinded to the treatment allocation.

### Primary outcome

The primary outcome is the FMA-UE, which has been developed as an evaluative measure of the upper limb functionality recovery poststroke (25–27). The FMA-UE score is 66 points, with lower scores indicating more severely impaired UE and higher scores indicating better motor function improvements. The baseline assessment results of this study are recorded as FM0, the second assessment results are carried out as FM1 after interventions, and the follow-up assessment results are recorded as FM2.

### Secondary outcomes

#### Independence function and muscle strength

The effects of VR at the level of activity will be investigated. The Functional Independence Measure (FIM) is one of the secondary outcomes, which aims to quantify and measure whatever the individual personally achieves (28). It consists of 66 points, with lower scores denoting more severe UE impairment and greater scores suggesting better motor function gains. The Barthel Index (BI) is considered to measure independence in activities of daily living (ADLs) (29). It has a 100-point scale, with lower scores denoting serious ADL interdependence and higher scores suggesting improved ADL independence. A hydraulic Jamar hand dynamometer was used to measure the maximum grip strength. This was kept according to the protocol of the American Hand Therapy Association (30).

### Imaging outcomes

Secondary outcomes will be obtained through the use of rs-fMRI and ts-fMRI, which will be assessed to identify changes in the BOLD effect in ipsilesional and contralesional M1 on the left and right hemispheres, and also EEG. The MRI scanner is a 3.0T Philips (Philips Achieva, Philips, Medical Systems, Best, Netherlands) superconducting MRI system, and the coil used for scanning is a standard 32-channel head coil. Each subject will be laid supine, with the head in a neutral posture and secured with a belt and foam pads during the test. The scanning parameters are as follows: rs-fMRI: echo-planar imaging (EPI), the 46 layers of transverse fault, a layer of thickness of 2.5 mm, a layer spacing of 0.5 mm, the repetition time (TR) of 2,000 ms, the echo time (TE) of 20 ms, a tilt angle of 90°, the field of view (FOV) of 240 mm × 240 mm, and an acquisition matrix of 96 × 96. After the resting function scan, the task function scan under the same parameters will be performed, and the subject will be stimulated by the motion observation task during the ts-fMRI test.

A brain function audio-visual stimulation system (Samrtec SA-9900, Shenzhen Meide Medical Electronics Technology Co., Ltd., China) will be used to display the stimulation images, which should ensure synchronization between the picture stimulation task and scanning. The hemiplegic hand will be used by the subjects to make the conventional whole-hand fist closures. On the video screen, written instructions will be given for 2 s, instructing individuals to move the hemiplegic hand. The instruction text will be replaced with a black circle on a white screen that has begun blinking red at a rate of 2 Hz after a randomly jittered delay of 1.5–2.5 s. Subjects will be told to make fist closures at the same frequency that the red circle appeared.

The hands will move in full finger flexion and extension at the frequency of the visual cue while both hands are resting in a supine position on cushions next to the hips. After 30 s, the circle vanishes, and a white screen will instruct the subjects to put their hands down for ~30 s before the start of the following block of motions. In total, five stimulus events and four periods of resting time will total 270 s in each fMRI test.

Before scanning, subjects will undergo task familiarization training until a steady performance is attained. Patients who are unable to execute at the necessary frequency will be told to perform as closely as they can to the visual cue while maintaining proper fist opening and closing.

EEG detection (power spectrum and scalp function connection) will be carried out by Cadwell easy-type III EEG (Cadwell, Washington, USA) produced by Kaiwei Company in the United States. The participants will sit in a comfortable chair and wear an EEG hat that will be connected to the Cadwell Easy III recording system. EEG data will be recorded at a sampling rate of 500 Hz from 17 silver electrodes of Fp1, Fp2, F3, F4, F7, F8, T3, T4, C3, C4, Cz, T5, T6, P3, P4, O1, and O2, and the other two reference electrodes will be connected to the left and right auricles. Each electrode is filled with an electrode gel (Weaver and Company, Colorado, USA) produced by Weaver Medical Technologies to ensure good electrical contact between the electrode and the skin. The electrode impedance is maintained below 10 kΩ. During the experiment, participants will be instructed to keep their arms and hands relaxed. Throughout the experiment, the actual execution of hand movements will be prohibited due to large individual variations in the participants' level of motor control, and the information will be saved on a computer for further offline processing.

### Safety evaluation and adverse events

This method does not affect the normal physiological state. Tests for safety indicators before and after treatment include general physical examination (blood pressure, pulse, and respiration), electrocardiogram, blood routine, urine routine, stool routine, and liver and kidney function tests. Adverse events such as pain, dizziness, local infections, allergies, and seizure will be meticulously documented in the case report form (CRF). Serious negative events, such as fatalities or situations posing a serious risk to one's life, will be notified right away to the researcher and to the ethics committee within 24 h. The visual analog scale (VAS) will be assessed before and after intervention training and after fMRI and EEG to grade sleepiness, fatigue, and anxiety (31). In this case, the research team will give the patient treatment and recommendations based on the situation, evaluate whether he/she could continue to participate in the study, and award compensation in accordance with that decision.

### Data management

Skilled assessors will utilize CRFs to precisely collect patient data and information before importing them into an electronic database. The paper CRFs will be maintained in a locked cabinet when the study is finished. Researchers never change the data because it has already been secured in the electronic database as well. Personal information about participants will be kept anonymous and under tight confidentiality. With their approval, we will collect patient data over the phone for those who drop out or withdraw from the study in the experiment.

### Data analysis

Data analysis will be performed using SPSS software (IBM, Statistics 25 version). Measurement data that follow a normal distribution as a whole will be expressed as mean ± standard deviation (X ± SD), and the whole data will not follow a normal distribution in median (interlude spacing) [M(P25–P75)]. The count data will be expressed as the number of cases (composition ratio) [*N*, (%)], and the analysis is tested by the *X*^2^ or Fisher's exact tests precisely. The comparison between the groups will be carried out by the *t*-test or the Mann–Whitney *U*-test based on whether the measurement data present a normal distribution and homogeneous variance to compare the differences in FMA-UE and independent sample *t*-test for the parameter in FIM, BI, grip strength, fMRI and EEG at a *p*-value below 0.05. There are no interim analyses planned.

fMRI image processing will use the statistical parameter graph software statistical parametric mapping (SPM)12. Before data processing, all data will be adjusted in orientation; and then head-action alignment, high-resolution structural image, and the average functional image will be registered; the high-resolution structural image after registration will be split; all the aligned functional images will be standardized to the Montreal Neurological Institute space using the standardized parameters generated during the segmentation process; the standardized voxel size is 3 mm × 3 mm × 3 mm; and the standardized functional image is spatially smoothed. Through an intragroup analysis, a paired *t*-test will be performed to assess brain alterations in each group (before and after the treatment). Age and gender will be taken into account as variables in the data analysis. The association between the improvement value of the correlation scales and the change in fMRI image data will be examined using the Pearson correlation coefficient.

For pre-processing and analysis, EEG data will be exported to MATLAB 9.2.0 (MathWorks, Inc., Natick, MA). A second-order Butterworth filter with a bandpass of 0.1–70 Hz and a band stop of 49–51 Hz will be used to filter the data. The data will be divided into 90 segments of 2 s each, and epochs with obvious artifacts will be removed by visual inspection. To find and eliminate non-physiological artifact components, data will be subsequently subjected to an independent component analysis using the EEGLAB rapid independent component analysis (ICA). The missing channels will be interpolated after the artifacts will be removed. The frequency domain indicators will be extracted and counted, Fourier transform on the preprocessed data will be performed and converted into a power spectral density (PSD) in decibels, each frequency band of interest will then be exported, the power value will be calculated, statistical analysis will be performed, and the difference will be output into the document.

### Quality control

The reasons for the suspension of the study and the relationship between the clinical study should be carefully recorded, and the criteria for the suspension of clinical research should be stipulated. When the subject will suspend the clinical study, the corresponding clinical evaluation will be carried out, and the statistical processing principle of the data after the suspension of the clinical study will be determined. For the subject who proposes to withdraw from the clinical study in the middle, the reason should be clearly recorded, if the subject will not be followed up on time, the reason should be consulted by telephone or letter, and the situation after the study will be investigated, and the clinical evaluation of the subject when withdrawing from the clinical study will be carried out.

In order to make this project function smoothly, the relevant operating procedures of clinical research and the implementation process will be scientifically and rigorously standardized. Researchers will be trained uniformly to master the implementation of interventions in the intervention group and the control group so as to improve the internal observation consistency and observer-to-observer consistency of researchers and ensure the reliability of clinical research conclusions. Quality monitors will be set up to carry out quality control and supervision of the whole process of research, the records and reports of all research data will be checked and confirmed, and the correctness and completeness of the case report will be formed, and it will be ensured that they are consistent with the original data. The trial review team consists of two staff members from the review department and will be responsible for a regular review of progress, authenticity, and safety every 12 weeks. Trial audit teams, independent data monitoring, and ethics committees must review the conduct throughout the trial.

## Discussion

Extensive studies have been published demonstrating the effectiveness of VR training and related therapies in enhancing upper motor function, with combination therapy appearing to be more beneficial (32, 33). Furthermore, they continue to have a limited scope, focusing just on the effectiveness without delving into the process. Based on our prior research, the neuroplasticity hypothesis, and real-world experience, VR training could be a multimodal therapy to induce cortical reorganization in stroke patients.

At present, no accepted theory could explain the neural mechanism of VR to promote motor function recovery, and the study of interhemispheric inhibition models has been relatively perfect (34), but alternative models and related influencing factors, such as synaptic regeneration ability, still have large research space in patients with various stages of stroke (35). VR is a rehabilitation treatment technology with great development potential, with the deepening of research study and the rapid development of technology, and it is likely to occupy a larger application space and a more important position in stroke upper extremity rehabilitation. Therefore, the underlying neural mechanisms of the impact of VR use on stroke rehabilitation still need to be further studied. How VR induces activation in adjacent cortical regions and whether the brain could produce effective synapses through motor learning and high-intensity training need further verification.

Research by Garcia et al. (36) showed that VR training effectively promoted the functional remodeling of the brain. Some studies have also found that VR training can activate the motor cortex, cerebellar cortex, and frontal white matter areas of the patient's contralateral side (37, 38). When completing virtual activities, visual and auditory feedback can enhance the motivation and focus of the participants, thereby improving the patient's attention and spatial perception function (39). By repeating the virtual activity task, the participants' motor learning ability and executive function can be strengthened (40). In addition, studies have confirmed that exercise training itself can also promote the recovery of cognitive function in patients after stroke, and the reason may be that exercise can promote the growth and survival of neurons in the affected side of the brain (41). A total of two studies reported clinical outcomes of improved upper extremity motor function after VR rehabilitation, with higher fractional anisotropy (FA) in the ipsilateral corticospinal tract (CST) and transcallosal motor fibers in a bilateral arm training study (42) and increased bilateral sensorimotor cortex and hippocampal gray matter volume in a constraint-induced movement therapy study (43). A previous study of patients with subacute stroke showed an initial decrease in FA in the ipsilateral CST and transcallosal M1–M1 fibers in the early post-stroke period (44).

A single modality of neuroimaging has been unable to meet the needs of researchers, who hoped to achieve multimodal observation of the brain by integrating information from both temporal and spatial aspects of the brain. Neurological imaging tended to obtain information from multiple modalities and integrate them and combined with different advantages of modal imaging technology so as to obtain more comprehensive, rich, accurate, and reliable brain information, and this method also provides a new monitoring method for the description of the cognitive neural dynamic process (45). VR-associated neurophysiological changes can be assessed by non-invasive and portable neuroimaging techniques including fMRI (46) and/or EEG (47) to determine changes in cerebral hemodynamic responses or oscillating brain waves, respectively. In particular, the use of fMRI as a measure of cerebral hemodynamic response to the neurological rehabilitation process has received attention. In addition, EEG has long been used to measure brain activity levels during cognitive or motor tasks and in a variety of clinical populations. The cortical activation measurements in fMRI and EEG or functional near-infrared spectroscopy (fNIRS) techniques show a high degree of correlation between motor and cognitive tasks (48, 49). Above all, valid methodologies to correlate objective functional measures that are valid and repeatable are the brain plasticity measure techniques using the EEG. The previously stated techniques can help rehabilitation specialists fully comprehend how such a patient's temporal and spatial neural plasticity keeps changing after VR intervention, allowing them to track the progress of patients in recovery, ascertain their reactions, and adjust the training modules to fit their unique program. The ability of fMRI and EEG to detect changes in neurophysiological measurements can provide feedback on where and how well the brain is activated, and clinical researchers can use this feedback to set treatment intensity and adjust the next treatment plan. Therefore, observing neurological defects of M1 through fMRI and EEG is of certain significance to explore the mechanism of VR in the treatment of stroke.

In this study, the FMA-UE score will be used to explore the behavioral changes of VR intervention combined with conventional rehabilitation training to improve the motor function of the upper extremities on the hemiplegic side. As previously stated, VR-based exercise therapy may help improve FMA-UE, allowing patients to engage more actively in the activities daily of living while also demanding less support from healthcare professionals or caretakers. In order to predict stroke early, the regional reconstruction of cerebral cortex function and activation of brain regions with quantitative parameters is evaluated and the correlation between the application value and motor function of combined metabolic functional imaging index and electroencephalographic imaging index in the evaluation of stroke patients before and after VR rehabilitation is explored. The upper extremity motor function of patients will be provided with quantitative indicators, trying to find out the patient group with potential benefits of rehabilitation therapy and guide the clinical practice of reasonable and effective VR rehabilitation therapy.

## Ethics statement

The studies involving human participants were reviewed and approved by the Ethics Committee of the Second Hospital of Jiaxing (reference number: JXEY-2023SW036). Written informed consent to participate in this study was provided by the patients/participants.

## Author contributions

JS, XG, and JF contributed to the conception and design of the study. JS wrote the first draft of the manuscript. YY and CL revised this manuscript and conducted the preliminary pre-experiment. YL and ZL recruited patients and provided treatment. MZ conducted a statistical analysis in the pre-experiment. All authors contributed to the manuscript revision, read, and approved the submitted version.
